# Feeding a Novel Mannan-Rich Yeast Carbohydrate Product Improves Production Performance and Humoral Immunity of Broiler Chickens

**DOI:** 10.3390/ani14111667

**Published:** 2024-06-03

**Authors:** Anhao Wang, Anangelina Archile, Rob Patterson

**Affiliations:** CBS Bio Platforms, 4389-112 Ave SE, Calgary, AB T2C 0J7, Canada; aarchile@canadianbio.com (A.A.); rpatterson@canadianbio.com (R.P.)

**Keywords:** mannan-rich yeast carbohydrate, broiler chicken performance, antibody response

## Abstract

**Simple Summary:**

As demands for high-quality protein like chicken meat constantly rise, producers and researchers are actively looking for ways to continuously improve chicken health and performance. The present study examined the effectiveness of a novel mannan-rich yeast carbohydrate product to improve chicken growth performance and immunity. We demonstrated that supplementing mannan-rich yeast carbohydrate improved broiler chicken feed efficiency and antibody immune responses. Our study suggested that the novel mannan-rich yeast carbohydrate product has significant potential for use in poultry production.

**Abstract:**

The current study examined the benefits of a novel mannan-rich yeast carbohydrate product (YM) on broiler chicken growth performance and immune response against sheep red blood cells (SRBCs). A total of 144 newly hatched male Cornish cross broiler chicks were randomly assigned to four treatments with 12 cages per treatment and three birds per cage. The treatments were (1) control, basal diet; (2) YCW, basal diet + 1 g/kg yeast cell wall; (3) YM1, basal diet + 0.5 g/kg of a novel yeast mannan-rich product (YM); and (4) YM2, basal diet + 1 g/kg YM. Growth performance was measured at 14, 28, and 35 days of age (d). At 26 and 27 d, nine birds per treatment were immunized intravenously with SRBCs, and antibody responses against SRBCs were analyzed through a hemagglutination assay 7 days post-inoculation. Supplementing YM tended to improve broiler chicken weight gain from 29 to 35 d (*p* = 0.053). An improvement in the feed conversion ratio (FCR) was observed in the birds fed YM diets during 29–35 d and over the entire experimental period (0–35 d; *p* < 0.05). Furthermore, birds fed YM2 diets had more robust antibody responses against SRBCs than the control birds (*p* = 0.033). In conclusion, dietary supplementation of YM improved broiler chicken growth performance and antibody response against SRBCs.

## 1. Introduction

Yeast and its derivative products have long been used in animal agriculture as nutrient sources or as feed additives that improve animal health and performance [1,2,3]. Among the yeast derivative products, the yeast cell wall and its bioactive components, including mannans and β-glucans, have drawn significant research interest and shown promising benefits such as enhancing immune functions, promoting gastrointestinal mucosa development, and improving production performance [2]. However, comprehensive literature reviews indicated that the effects of dietary supplementation of yeast cell wall and its derivative products are inconsistent [2,3,4,5,6]. Meta-analysis [4,5,6] demonstrated that more than 70% of studies showed the benefits of dietary supplementation of yeast cell wall and accompanying derivatives, while the remaining studies indicated these feed additives did not improve or even compromised animal performance. The inconsistency observed between studies could be attributed to various factors, such as the strain of origin, physical properties (e.g., water solubility), and downstream processing, as well as dosage, animal health status, and management practices [7,8].

Mannans are water-insoluble long-chain polysaccharides and are major yeast cell wall components [2,3]. More specifically, mannans compose around 41% of the non-starch polysaccharide (NSP) fraction of the yeast cell wall, while β-glucans compose around 58% of the yeast cell wall NSP [2]. It should be noted that the yeast cell wall mannan/glucan ratio can vary greatly among different strains [2]. The mannan fractions of the yeast cell wall have immunomodulatory properties and could be used to maintain and/or promote growth performance, health, and immunity in broiler chickens [9]. In recent studies, supplementing mannan-rich yeast products improved both broiler chicken and laying hen production performance and immune responses [10,11]. However, similar to yeast cell wall products, supplementing yeast mannan products did not consistently improve broiler chicken performance and health parameters [3,12]. The inconsistent outcomes could be due to factors such as the insolubility of mannans in the form they are fed to animals and variability in the amount of mannans fed. Conversely, positive results that have been observed could be attributed to mannans when, in fact, the preparation likely contained β-1,3-glucans. Therefore, in order to elucidate the mode of action associated with dietary supplementation of yeast mannans, it is necessary to develop a yeast mannan preparation that can consistently improve broiler chicken performance and immune parameters.

The objectives of the present study were to evaluate a novel mannan-rich yeast carbohydrate product (YM) on broiler chicken growth performance and immune response following the immunization with sheep red blood cells (SRBCs). The current study will also collect baseline data on feeding a novel yeast supplement that contained a relatively high mannan to glucan ratio to better understand the mode of action of such a preparation within the diet of broiler chickens.

## 2. Materials and Methods

### 2.1. Animals and Dietary Treatment

All procedures followed the recommendations of the Canadian Council on Animal Care [13] and the National Farm Animal Care Council Code of Practice [14]. The mannan-rich yeast supplement was prepared by enzymatic hydrolysis at CBS Bio Platforms Inc. (Calgary, AB, Canada). Briefly, the yeast cell wall (*Saccharomyces cerevisiae*) was suspended in water (1:3 *wt*/*wt* ratio) and subjected to a proprietary multi-enzyme preparation. The solution was incubated at 40 °C for 48 h under continuous mixing at 200 rpm using an overhead stirrer. Immediately after the incubation, the solution was heated at 60 °C for 10 min in a water bath to inactivate the enzymes. Then, the solution was spray-dried. The mannan/glucan ratio (M/G ratio) of the yeast cell wall (YCW) and the enzymatically treated product was determined by gas-liquid chromatography at the University of Manitoba (Winnipeg, MB, Canada). The M/G ratio of untreated YCW was 1:1, and the enzymatically treated product had an M/G ratio of 10:1. Thus, the enzymatically treated product was referred to as novel mannan-rich yeast carbohydrate (YM).

One hundred and forty-four newly hatched male Cornish cross broiler chicks were obtained from a local commercial hatchery. Birds were randomly assigned to 48 battery cages (38 cm high × 41 cm wide × 51 cm long; providing 697 cm^2^/ bird floor space) with 3 birds/cage in a temperature-controlled room at the Poultry Research Unit of CBS Bio Platforms (Calgary, AB, Canada). There were 4 treatments, and each treatment was replicated 12 times. The dietary treatments consisted of (1) control, basal diet; (2) YCW, basal diets + 1 g/kg YCW; (3) YM1, basal diets + 0.5 g/kg YM; and (4) YM2, basal diets + 1 g/kg YM. The basal diets were corn and soybean meal-based and were in mash form. The basal diets were formulated to meet or exceed the nutrient requirements of broiler chickens as recommended by the National Research Council [15] and were manufactured at a commercial feed mill (Country Junction Feeds, Wetaskiwin, AB, Canada). The diet formulations and calculated and analyzed nutrient compositions of the basal diets are shown in Table 1.

### 2.2. Growth Performance

Broiler body weight (BW, g) was recorded at 14, 28, and 35 d. Feed consumption was measured at 14, 28, and 34 d or as mortality occurred. The average daily gain (ADG, g/d), average daily feed intake (ADFI, g/d), and feed conversion ratio (FCR, g feed/g weight gain) of each cage were then calculated.

### 2.3. Inoculation and Antibody Titration

At 26 and 27 days of age, a total of 12 birds per treatment were randomly selected to assay T-cell-dependent antibody responses. Sheep red blood cells (SRBCs, Innovative Research Inc., Novi, MI, USA) were used as the antigen. The procedures were previously described [16,17,18,19]. Before immunization, approximately 2 mL of blood per bird was collected to determine baseline antibody responses. Nine birds per treatment were immunized intravenously through the brachial vein with 1 mL of 2% SRBCs in phosphate-buffered saline (PBS). Additionally, 3 birds per treatment were sham-injected with 1 mL of PBS. At 7 days post-immunization (33 and 34 days of age, respectively), around 2 mL of blood per bird was collected from immunized and sham-injected birds. After blood collection, the samples were centrifuged at 580× *g* for 10 min to isolate plasma. The plasma samples were stored at −20 °C until antibody analysis. Detection of the total antibody response to SRBCs in plasma was performed by a direct hemagglutination inhibition assay. Prior to analysis, samples were heat treated at 56 °C for 30 min. Fifty μL of PBS was added into each well of a U-bottomed 96-well microplate. Then, 50 μL of plasma samples were added into the first column of the plate and serially double diluted within each well. Subsequently, 50 μL of 2% SRBCs in PBS was added to each well, and the plates were shaken for 1 min followed by incubation for 3 h at 37 °C. A positive result for agglutination titer was recorded at the first dilution with teardrop formation of SRBCs formed after tilting the plate.

### 2.4. Statistical Analysis

Statistical analyses were performed using the PROC MIXED procedure [20] of SAS On Demand (SAS Institute Inc., Cary, NC, USA). The experimental unit for performance was the cage, and for antibody titration analysis, the bird was used as the experimental unit. Significant treatment effects (*p* ≤ 0.05) and trends (0.05 < *p* ≤ 0.10) were analyzed using the Tukey–Kramer test to differentiate the means. Graphs were constructed using GraphPad Prism 10 (GraphPad Software Inc., Boston, MA, USA).

## 3. Results

During the starter (0–14 d) and grower (15–28 d) stages, growth performance did not differ among treatments (Table 1). During the finisher stage (29–35 d), YCW and YM 2x-fed birds tended to have higher ADG than the control with 101.3 and 101.7 g/d compared with 86.6 g/d, respectively (*p* = 0.053; Table 2). Throughout the experimental duration, the ADFI was not affected by the treatments (*p* > 0.10, Table 2). Meanwhile, the YM2-fed birds had lower FCR than the control birds during 29–35 d and through the entire experimental duration (*p* < 0.05, Table 2). The overall mortality for the entire flock was 2.8% (4 birds) during the experimental duration and did not differ among treatments.

**Figure 1:**
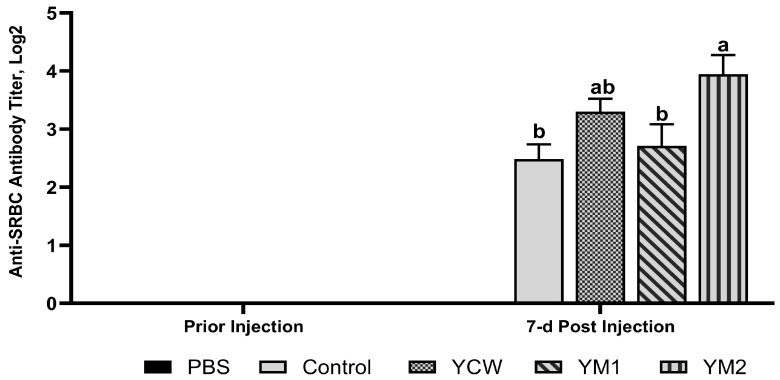
Effects of yeast cell wall (YCW) and a novel mannan-rich yeast carbohydrate (YM) supplementation on broiler chicken antibody response against sheep red blood cells (SRBCs). The SRBC-specific antibody was determined by hemagglutination assay. Bars represent means ± SEM with *n* = 9 animals. The bars with different letters a and b differed significantly (*p* = 0.033, one-way ANOVA followed by Tukey–Kramer test, *p* ≤ 0.05). The prior to injection and PBS-injected birds did not show SRBC-specific antibody responses. Control: basal diet; YCW: basal diets + 1 g/kg yeast cell wall (YCW, CBS Bio Platforms Inc., Calgary, AB, Canada); YM1: basal diets + 0.5 g/kg YM and YM2: basal diets + 1 g/kg YM.

With respect to the SRBC antibody response, birds fed the YM2 diets had higher SRBC-specific antibody titers than the control and YM1 birds (*p* = 0.033, Figure 1). Furthermore, YCW-fed birds’ SRBC-specific antibody titers differed from neither the control nor the YM2 birds (Figure 1). As expected, the birds did not show SRBC-specific antibody responses before immunization and after sham PBS injection (Figure 1).

## 4. Discussion

The current study demonstrated that dietary supplementation of a novel mannan-rich yeast carbohydrate preparation improved broiler chicken growth performance. The growth enhancement observed in the current study was in line with previous meta-analyses, which indicated that dietary supplementation of yeast mannan products improved broiler chicken weight gain and feed efficiency [4,5,6]. More specifically, we demonstrated that the most pronounced improvement in performance was observed in the birds fed YM diets during the later stage of growth. Additionally, our findings agreed with recent studies, which also reported improved growth performance observed in birds that were fed yeast mannan-supplemented diets during the later stages of growth [8,21].

Previous studies that examined the effects of dietary yeast carbohydrate supplementation did not consistently improve bird performance. This lack of response can be attributed to the fact that no external immune challenge such as *Clostridium perfringens*, *Salmonella enteritidis*, or *Eimeria* spp. was applied [2,3]. However, when improved growth performance did occur, the beneficial effects of yeast mannan products were frequently observed when the supplementation rate exceeded 1 g per kg of diet [8,22,23]. In contrast, in those studies, yeast mannan products did not improve production performance when included at levels less than 1 g per kg of diet. In line with the current study, Pascual et al. [21] also reported that dietary supplementation at a rate of 0.5 g per kg with an alternate yeast mannan preparation improved broiler chicken FCR during later stages of growth and over the entire 42-day rearing period. The different inclusion rates of a specific yeast mannan product required to achieve enhanced performance in broiler chicken feeding studies could be due to factors such as diet formulations and rearing conditions, as well as the nature of the products such as differences in the origin strain and the preparation methods used during product manufacturing [2,7,24]. A recent study demonstrated the importance of the preparation method and the efficacy of specific yeast cell wall carbohydrate preparation to mitigate the adverse effects of *S. enteritidis* [25]. In the in vitro phase of that study, the specific enzymatic treatment improved the water solubility of yeast cell wall carbohydrates. Moreover, in the subsequent in vivo phase of the study, the yeast carbohydrate preparation that was enzymatically treated and was highly water-soluble demonstrated a greater ability to reduce *S. enteritidis* shedding in laying hens than the intact, less water-soluble counterpart [25]. The concept of increasing the water solubility of yeast cell wall components through enzymatic lysis has long been proposed [26]. Recent studies demonstrated that dietary supplementation of enzyme-modified yeast carbohydrate products provided a wide range of beneficial effects in poultry, such as promoting intestinal mucosa structure development, mitigating the adverse effects of pathogens, and enhancing innate and humoral immune responses, as well as improving performance [8,17,27,28,29,30,31]. Water solubility is considered a critical parameter for yeast carbohydrates to exhibit the aforementioned beneficial effects [2]. Therefore, the different supplementation rates required to achieve performance effects in the current and previous yeast mannan studies [21,22,23] could be due to differences in water solubility of the various yeast mannan products evaluated in the different studies. Thus, determining the product’s water solubility could be used as a screening method in future studies that assess the benefits of supplementing various yeast mannan preparations in vivo.

In addition to determining growth performance, the effects of dietary YM supplementation on broiler chicken immune functions were assessed using SRBCs as a noninfectious antigen. The results demonstrated that supplementing 1 g per kg of YM improved the primary antibody response, immunoglobulin M (IgM), against SRBCs. This finding supports previous studies where chicken antibody responses were strengthened by dietary supplementation of a preparation that contained yeast cell wall carbohydrates [32,33]. In the current study, the more robust antibody responses observed could be the results of immune-modulating effects associated with yeast cell wall-derived preparation. For example, in a recent study, a chicken B cells line (DT40) was used as a model to examine the potential mechanisms of immune modulation associated with various yeast carbohydrate products [28]. When the experimental cells were subjected to an *E. coli* lipopolysaccharide (LPS), DT40 cells exposed to an enzymatically treated yeast carbohydrate increased the gene expression of key immune-modulating cytokines, such as toll-like receptor 2b (TLR2b), interferon-gamma (INF-ɤ), and interleukin 4 (IL-4) and 12 (IL-12) as compared with the control challenged cells or challenged cells that co-incubated with the intact yeast cell wall [28]. Those aforementioned cytokines have critical immune-strengthening functions such as detecting antigens, as well as promoting growth, differentiation, and functionality of B and T cells [2]. The in vitro immune modulation effects highlighted by Echeverry et al. [28] may explain the underlying mechanisms of the stronger antibody response against SRBCs observed in the YM-fed birds in the current study.

Using SRBCs as an antigen in concert with a hemagglutination detection method to evaluate T-cell-mediated antibody responses in poultry is well established and frequently used [16,34,35]. In the current study, a similar immunization program to that used by Singh et al. [36] was applied, where broiler chickens were only subjected to one SRBC immunization. However, in the majority of studies where SRBCs were used to evaluate an immune response in chickens, two immunizations were conducted to evaluate the primary (IgM) and secondary (IgY) antibody responses [16,17,33,35,37]. The secondary antibody response is critical for animals to maintain long-term immunity and health as it can rapidly and effectively eliminate the previously exposed antigens [38]. In future studies, two SRBC immunizations will be conducted to fully assess the effects of YM on the immune response of broiler chickens.

Other beneficial effects of yeast mannan products, such as enhancing gastrointestinal (GIT) mucosa structure development, increasing nutrient digestibility, and reducing the impacts of *E. coli* LPS and *Salmonella enteritidis* colonization have been reported [21,22,25,29,32,39,40] but were not determined in the current study. In future studies, the effects of dietary supplementation of YM on broiler chicken GIT development, nutrient digestibility, and GIT microbiota compositions will be determined to provide a more comprehensive assessment of the benefits of this novel mannan-rich yeast carbohydrate product on broiler chickens.

## 5. Conclusions

The current study demonstrated that dietary supplementation of a novel mannan-rich yeast had multiple beneficial effects, such as improved broiler chicken growth performance and immune response. Further research is required to thoroughly examine the effects of this novel mannan-rich yeast carbohydrate on GIT development, immune function as well as the different application strategies in both healthy birds and birds that are subjected to challenged conditions, such as heat stress, feed restrictions, and pathogen challenges.

## Figures and Tables

**Table 1:** Feed formulation and calculated and analyzed nutrient compositions of the basal diets.

Item	Starter (0–14 d)	Grower (15–28 d)	Finisher (29–35 d)
Ingredient, %			
Corn	50.36	47.57	55.82
Soybean meal, 47.5% CP	29.89	22.27	19.97
Canola meal	8.00	10.00	9.14
Wheat	7.20	14.50	10.00
Faba bean	0	0.65	0
Dicalcium phosphate	1.63	0.87	0.72
Limestone	0.96	1.07	1.41
Canola oil	0.50	1.75	1.74
L-Methionine	0.33	0.25	0.22
Salt	0.29	0.29	0.29
Lysine-HCl	0.29	0.26	0.23
L-threonine	0.09	0.06	0.03
Sodium bicarbonate	0.15	0.15	0.15
Limestone	0.96	1.07	1.41
Canola oil	0.50	1.75	1.74
L-Methionine	0.33	0.25	0.22
Salt	0.29	0.29	0.29
Lysine-HCl	0.29	0.26	0.23
L-threonine	0.09	0.06	0.03
Sodium bicarbonate	0.15	0.15	0.15
Mineral premix ^1^	0.10	0.10	0.10
Vitamin B premix ^2^	0.05	0.04	0.04
ADE vitamin premix ^3^	0.04	0.03	0.03
Selenium 1000 mg/kg	0.03	0.03	0.03
Rovimix HyD Premix ^4^	0.02	0.06	0
Vitamin D—10,000 KIU/kg ^5^	0.02	0	0.03
Choline chloride 60%	0.02	0	0
Tribasic copper chloride ^6^	0.01	0.01	0.01
Vitamin E 50% ADS ^7^	0.01	0.01	0.01
Superzyme^®^-W Conc ^8^	0.02	0.02	0.02
Bio-Phytase 5000G ^9^	0.01	0.01	0.01
Analyzed Nutrient Composition ^10^			
Crude protein, %	24.3	21.5	19.1
AME, kcal/kg	3018	3078	3120
Calcium, %	0.96	0.75	0.87
Phosphorus, %	0.86	0.65	0.56
Crude fiber, %	2.32	2.34	2.32
Fat, %	3.18	4.43	4.52
Ash, %	5.77	4.43	5.03
Calculated amino acid, %			
Lysine	1.39	1.23	1.12
Methionine	0.63	0.55	0.51
Methionine + cysteine	1.04	0.94	0.87
Threonine	0.95	0.84	0.76

^1^ Mineral premix: provides 110,000 mg/kg Zn, 130,000 mg/kg Mg, 65,000 mg/kg Fe, 20,000 mg/kg Fe, 2500 mg/kg I, and 500 mg/kg Co (Country Junction Feeds, Wetaskiwin, AB, Canada). ^2^ Vitamin B premix: provides 80 mg/kg cobalamin (vitamin B12), 10,000 mg/kg menadione (vitamin K3), 1200 mg/kg biotin (vitamin B7), 4500 mg/kg folic acid (vitamin B9), 120,000 mg/kg niacin (vitamin B3), 35,000 mg/kg pantothenic acid, 28,000 riboflavin (vitamin B6), and 6500 mg/kg thiamine (vitamin B1) (Country Junction Feeds, Wetaskiwin, AB, Canada). ^3^ ADE vitamin premix: 30,000 KIU/kg retinol (vitamin A), 3000 KIU/kg cholecalciferol (vitamin D3), and 100,000 IU/kg α-tocopherol (vitamin E) (Country Junction Feeds, Wetaskiwin, AB, Canada). ^4^ Rovimix HyD Premix: 5512 KIU/kg (DSM Nutritional Products Canada Inc., Mulgrave, NS, Canada). ^5^ Vitamin D—10,000 KIU/kg: 10,000 KIU/kg (Country Junction Feeds, Wetaskiwin, AB, Canada). ^6^ Tribasic copper chloride: 1000 mg/kg Cu (Micronutrients USA LLC, Indianapolis, IN, USA). ^7^ Vitamin E 50% ADS: 500,000 IU/kg α-tocopherol (Shandong NHU Vitamin Company Ltd., Yangzi, Shandong, China). ^8^ Superzyme^®^-W Conc: 1000 units/g glucanase, 8000 units/g xylanase, 5000 units/g cellulase, 14,000 units/g amylase, 400 units/g invertase, and 2600 units/g protease (CBS Bio Platforms Inc., Calgary, AB, Canada). ^9^ Bio-Phytase 5000G: 5000 units/g phytase (CBS Bio Platforms Inc., Calgary, AB, Canada). ^10^ Nutrient compositions were analyzed at Central Testing Laboratory Ltd., (Winnipeg, MB, Canada). AME = 53 + 38 × [(crude protein) + (2.25 × fat) + (100 − crude protein + crude fiber + fat + ash + moisture) × 0.9].

**Table 2:** Effects of yeast cell wall (YCW) and novel mannan-rich yeast carbohydrate product (YM) supplementation on broiler chicken performance.

Item	Control	YCW	YM1	YM2	*p*-Value
BW, g					
14 d	309.9 ± 7.5	302.6 ± 14.4	291.5 ± 11.3	286.4 ± 10.8	0.435
28 d	1246.4 ± 29.7	1245.1 ± 35.5	1224.6 ± 31.7	1258.9 ± 26.7	0.889
35 d	1852.8 ± 27.8	1964.9 ± 53.3	1894.8 ± 37.7	1971.2 ± 31.7	0.107
ADG, g/d					
0–14 d	19.7 ± 0.5	19.2 ± 1.0	18.5 ± 0.8	18.1 ± 0.8	0.498
15–28 d	67.0 ± 1.7	67.8 ± 2.0	66.1 ± 1.8	66.9 ± 2.0	0.945
29–35 d	86.6 ± 3.5 ^B^	101.3 ± 3.8 ^A^	95.8 ± 4.2 ^AB^	101.7 ± 5.3 ^A^	0.053
0–35 d	52.0 ± 0.8	55.2 ± 1.5	53.2 ± 1.1	55.4 ± 0.9	0.103
ADFI, g/d					
0–14 d	30.3 ± 0.8	28.9 ± 0.8	30.2 ± 1.2	30.0 ± 0.7	0.372
15–28 d	99.9 ± 1.6	98.2 ± 1.9	97.9 ± 2.7	95.1 ± 2.3	0.277
29–35 d	157.4 ± 9.5	157.2 ± 4.2	152.4 ± 4.0	154.6 ± 4.9	0.123
0–35 d	83.2 ± 1.2	82.3 ± 1.1	80.6 ± 1.5	82.2 ± 1.6	0.591
FCR, g/g					
0–14 d	1.550 ± 0.064	1.533 ± 0.074	1.645 ± 0.045	1.627 ± 0.071	0.603
15–28 d	1.497 ± 0.065	1.452 ± 0.024	1.471 ± 0.020	1.421 ± 0.031	0.746
29–35 d	1.727 ± 0.32 ^a^	1.556 ± 0.023 ^bc^	1.577 ± 0.053 ^bc^	1.518 ± 0.073 ^c^	0.041
0–35 d	1.604 ± 0.027 ^a^	1.522 ± 0.055 ^ab^	1.516 ± 0.019 ^ab^	1.486 ± 0.029 ^b^	0.008

In the same row, mean ± SEM with different superscripts a, b or c differ at *p* ≤ 0.05; means with different superscripts A and B differ at 0.05 < *p* ≤ 0.10 (one-way ANOVA followed by Tukey–Kramer test *p* ≤ 0.05; *n* = 12 per treatment). Control: basal diet; YCW: basal diets + 1 g/kg yeast cell wall (Maxi-Nutrio, CBS Bio Platforms Inc., Calgary, AB, Canada); YM1: basal diets + 0.5 g/kg novel mannan-rich yeast carbohydrate product; and YM2: basal diets + 1 g/kg novel mannan-rich yeast carbohydrate product.

## Data Availability

The data are contained within the article.
